# Predictors of facial attractiveness and health in humans

**DOI:** 10.1038/srep39731

**Published:** 2017-02-03

**Authors:** Yong Zhi Foo, Leigh W. Simmons, Gillian Rhodes

**Affiliations:** 1 ARC Centre of Excellence in Cognition and its Disorders, School of Psychology, University of Western Australia, 35 Stirling Hwy, Crawley, 6009, WA, Australia; 2 Centre for Evolutionary Biology & School of Animal Biology, University of Western Australia, 35 Stirling Hwy, Crawley, 6009, WA, Australia

## Abstract

Facial attractiveness has been suggested to provide signals of biological quality, particularly health, in humans. The attractive traits that have been implicated as signals of biological quality include sexual dimorphism, symmetry, averageness, adiposity, and carotenoid-based skin colour. In this study, we first provide a comprehensive examination of the traits that predict attractiveness. In men, attractiveness was predicted positively by masculinity, symmetry, averageness, and negatively by adiposity. In women, attractiveness was predicted positively by femininity and negatively by adiposity. Skin colour did not predict attractiveness in either sex, suggesting that, despite recent interest in the literature, colour may play limited role in determining attractiveness. Male perceived health was predicted positively by averageness, symmetry, and skin yellowness, and negatively by adiposity. Female perceived health was predicted by femininity. We then examined whether appearance predicted actual health using measures that have been theoretically linked to sexual selection, including immune function, oxidative stress, and semen quality. In women, there was little evidence that female appearance predicted health. In men, we found support for the phenotype-linked fertility hypothesis that male masculinity signalled semen quality. However, we also found a negative relationship between averageness and semen quality. Overall, these results indicate weak links between attractive facial traits and health.

Human facial attractiveness has been studied widely in the context of biologically-based preferences^1^,^2^,^3^. In contrast to the biological account, it has been suggested that our preferences are products of our cultural experiences^4^. This view has been challenged by two types of evidence. Not only is there cross-cultural agreement on what we consider attractive^5^,^6^, but also our facial preferences emerge at an early age^7^,^8^, before it is likely for cultural learning to have an influence on our preferences. Moreover, facial attractiveness and attractiveness-related traits have been shown to be associated with mate choice and mating success in humans^9^. Together, these results suggest that our facial preferences in part reflect adaptations that evolved via sexual selection.

Evolutionary theories propose that our preferences for certain traits evolved because those traits provide signals of biological quality, particularly physical health^10^. Numerous aspects of health, including immune function^11^, oxidative stress^12^, and semen quality^13^, have been associated with sexual signalling because of their importance to survival and/or reproduction. The immune system is the main physiological system for fending off disease-causing pathogens or parasites. Oxidative stress refers to the balance between the production of reactive oxygen species (ROS) that cause structural and DNA damage to cells and the efficiency of the antioxidant system to nullify the ROS^14^. Oxidative stress has been associated with diseases such as heart disease and cancer^15^,^16^. It is also linked to health functions such as semen quality and immune function due to the high concentration of polyunsaturated fats in sperm and immune cells, which make these cells highly susceptible to ROS damage^14^,^17^. Semen quality is important because sperm cells are critical for male fertility. Given the associations between the various aspects of health and survival/reproduction, it has been suggested that choosing a mate based on health can provide a variety of direct and indirect benefits^10^. Direct benefits include disease avoidance, increased fertility, and material advantages, such as better nutrition, parenting, and protection. Indirect benefits include obtaining genes that code for offspring health.

Evolutionary psychologists have identified several facial traits as potential candidates for biologically-based mate preferences, including sexual dimorphism (masculinity for men and femininity for women), averageness, symmetry, adiposity, and skin colour^1^,^2^,^3^. Sexual dimorphism, averageness, and symmetry, in particular, have been widely studied. A meta-analysis showed that these three traits are all significantly related to attractiveness^2^. Adiposity has also been found to be related to attractiveness^18^. The association between skin colour, particularly carotenoid-based skin yellowness, and facial attractiveness has been gaining attention only in recent years. Carotenoids are red and yellow pigments that influence our skin colour when consumed through fruits and vegetables. Inter- and intra-individual variation in fruit and vegetable intake correlates with skin yellowness^19^,^20^. Skin yellowness is related not only to facial attractiveness^21^, but also to other attractive traits such as sexual dimorphism^22^. In this study, we examine how all these traits are linked to attractiveness and health.

There are putative mechanistic links associating sexual dimorphism, averageness, symmetry, and skin colour with health. Male sexual dimorphism may signal health via the effects of testosterone^23^. Testosterone compromises health by suppressing immune function^24^ and increasing oxidative stress^25^. Therefore, theories suggest that only healthy males can afford to sustain elevated levels of testosterone for the development of sexually dimorphic traits^23^,^25^. Testosterone is also crucial for spermatogenesis^26^. Therefore, masculinity may also signal semen quality. Femininity has also been suggested to signal health in women via similar mechanisms with female hormones^2^,^27^, although there is some debate over the effect of female hormones on health^24^. Averageness and symmetry may reflect an individual’s ability to overcome the negative effects of genetic and environmental stressors during development^28^. Several mechanisms have been proposed as the link between carotenoid-based colouration and health. The carotenoid trade-off hypothesis proposed that using carotenoids for colouration prevents their use as antioxidants to quench ROS^12^,^29^. The hypothesis proposed that this trade-off leads to a significant relationship between colouration and health because healthy individuals can afford to devote more carotenoids to signalling. The hypothesis, however, has been questioned as some studies have failed to demonstrate that carotenoids possess antioxidant properties^30^, with a recent study finding that oral supplementation with the carotenoid beta-carotene did not affect health in humans^31^. As an alternative to the carotenoid trade-off hypothesis, the carotenoid protection hypothesis proposed that the carotenoid-based colouration could also signal the presence of other antioxidants that protect carotenoids from being damaged by ROS and losing their colour as a result^30^. Supportive evidence comes from findings that supplementation of non-pigmentary antioxidants enhanced carotenoid-based colouration intensity in a number of species^32^,^33^,^34^. To sum up, there are reasons to expect each of the traits to be correlated with health.

Although sexual dimorphism, averageness, symmetry, adiposity, and skin colour have all been associated with attractiveness, they have generally been studied individually. The exceptions were two recent studies which found that skin colour, specifically yellowness and lightness, predicted attractiveness in men while masculinity did not^21^,^35^. We still do not know much about the contributions of the different traits to attractiveness because no studies have examined them together.

Little is also known about the relationship between facial appearance and actual health. In general, theories of sexual signalling predict that attractive appearance would be positively related to actual health^10^,^11^,^13^. Only a few studies have examined the relationship between facial appearance and measures of immune function or oxidative stress. Using a hepatitis B vaccination protocol, Rantala *et al*.^36^ reported that men’s immune functioning was related positively to their facial attractiveness and facial masculinity, and negatively to adiposity. Using an index of oxidative stress derived from measures of oxidative damage to the DNA and lipids, Gangestad *et al*.^37^ reported that oxidative stress was related to facial attractiveness, symmetry, and masculinity in men. These studies had only male participants, so it is also unclear whether the results hold for women. The few studies of the relationship between facial appearance and semen quality have reported mixed results. Soler *et al*.^38^ found that semen quality was positively related to facial attractiveness and negatively related to a masculine trait of the face: the width. In contrast, Peters, Rhodes, and Simmons^39^ did not find any relationship between semen quality and facial attractiveness or attractive traits such as masculinity, symmetry, and averageness. Notably, no studies have examined the relationship between any actual health measures and skin colour.

The aim of the present study is to provide a comprehensive assessment of the facial appearance predictors of attractiveness, perceived health, and actual health. First, we investigate whether any of the facial traits, including sexual dimorphism, averageness, symmetry, adiposity, and skin colour, positively predict facial attractiveness. Second, we investigate whether any of the facial traits positively predict perceived health. Lastly, we investigate whether any of the facial traits predict any of the actual health measures, including immune function, oxidative stress, and semen quality. With the exception of semen quality, we examine all relationships for both men and women.

## Results

Zero-order correlations between the appearance and health variables are presented in Table 1. For each sex, we ran separate multiple regression analyses to examine the facial appearance variables that predicted attractiveness, perceived health, and each of the actual health variables. We ran separate multiple regression models for colour and the other appearance traits. We did so because perception of sexual dimorphism, averageness, symmetry, and adiposity could be influenced by both face shape and colour. Indeed, the zero-order correlations show that skin colour is related to traits such as male symmetry and female femininity (Table 1). Therefore, analysing skin colour together with the other appearance traits in the same multiple regression models might lead us to underestimate the contribution of colour. Age was included as a control variable in all regression models. Visual examination of the scatterplots indicated a potential curvilinear relationship between adiposity and attractiveness in women. Therefore, we also added the quadratic term of adiposity into the model for female attractiveness.

### Facial attractiveness

Descriptive statistics for the facial appearance variables are presented in Table 2. According to the multiple regression results (Table 3), male attractiveness was significantly and positively predicted by masculinity, symmetry and averageness and negatively by adiposity. Female attractiveness was significantly and positively predicted by femininity and negatively by adiposity.

### Perceived health

Male perceived health was positively predicted by averageness, symmetry, and yellowness and negatively by adiposity (Table 4). Female perceived health was positively predicted by femininity (Table 4).

### Immune function

Principal components analyses (PCA) were conducted to summarize the interrelated immune function variables. Both male and female data returned two PCs (see supplementary material for details on data reduction). For men, PC1 was loaded mainly by bacterial killing capacity and overall bacterial immunity and PC2 was loaded mainly by bacterial suppression capacity and lysozyme activity. For women, PC1 was loaded mainly by bacterial killing capacity, overall bacterial immunity and lysozyme activity and PC2 was loaded mainly by bacterial suppression capacity. Residuals were extracted from women’s PC1 after controlling for significant lifestyle factors (see supplementary material). No lifestyle factors were significantly related to either PC2 in women or any of the PCs in men. Therefore, we ran these analyses on the raw data.

Multiple regression results indicated that there were no significant appearance predictors of either immune PC in either men or women (Tables 5 and 6).

### Oxidative stress

Raw descriptive statistics for the oxidative stress variables are presented in Tables 7 and 8. The isoprostane data was log-transformed to achieve normal distribution for the data analyses. The 8-OHdG analyses for women were based on residuals that were extracted after controlling for various lifestyle variables (see supplementary material). Lifestyle factors did not significantly affect isoprostane levels in women or either of the oxidative stress measures in men. Therefore, we ran the regression analyses on the raw data of these variables.

In men, multiple regression results indicated no significant appearance predictors of either oxidative stress measure (Tables 7 and 8). In women, there were no significant predictors of either oxidative stress measure (Tables 7 and 8).

### Semen quality

PCA was conducted on the interrelated semen quality variables, resulting in three PCs (see supplementary material). PC1 was weighted most strongly by variables related to rapid progressive motility. PC2 was weighted most strongly by variables related to the linearity of the sperm movement. PC3 was weighted most strongly by sperm concentration and percentage motile sperm. PC2 was reverse-scored, square-root-transformed, and then reversed-scored again to achieve normal distribution due to a negative skew and positive kurtosis. Residuals were extracted from PCs 1 and 2 after controlling for variation in lifestyle, collection procedure, and sample abnormalities (see supplementary material).

Multiple regression results indicated that PC1 was negatively predicted by skin yellowness (Table 9). PC 2 was positively predicted by masculinity (Table 10). PC3 was negatively predicted by averageness (Table 11).

## Discussion

This study provides a comprehensive assessment of the facial traits that predict attractiveness, perceived health, and various measures of actual health, including oxidative stress, immune function, and semen quality. Our results showed that male attractiveness was predicted positively by masculinity, symmetry and averageness, and negatively by adiposity. Female attractiveness was predicted positively by femininity and negatively by adiposity. Male perceived health was predicted positively by averageness, symmetry, and skin yellowness and negatively by adiposity. Female perceived health was predicted positively by femininity. In terms of actual health, there was little evidence that female facial appearance signalled health. In men, semen quality was positively predicted by masculinity, suggesting that masculinity may be a signal of male fertility.

Consistent with previous studies, our findings demonstrate the importance of sexual dimorphism, symmetry, averageness, and adiposity in determining attractiveness^2^,^18^. Sexual dimorphism, symmetry, and averageness have often been studied in terms of face shape. However, these traits can be influenced by colour as well^22^,^40^. Indeed, we found that redness was related to femininity in women. In men, lightness and yellowness were related to symmetry. It is possible, therefore, that the relationship between attractiveness and traits such as sexual dimorphism, symmetry, and averageness were partly accounted for by variations in skin colour. However, we did not find any significant relationships between skin colour and attractiveness in either sex. The null findings suggest that, despite recent interest in skin colour as a potential sexual signal, skin colour might play limited role in facial attractiveness. Consequently, it is unlikely that the observed relationships between attractiveness and sexual dimorphism, symmetry, and averageness were due to variations in skin colour.

Earlier studies examining the relationship between skin colour and attractiveness have used morphed images that were manipulated to vary in skin colour, finding that increasing yellowness increased attractiveness^19^,^20^. Although such studies have the advantage of manipulating specific facial features while holding all others constant, it is important to know whether naturally occurring variation in skin colour, both within and between individuals, is related to attractiveness. A recent placebo-controlled experimental study conducted by our group using the carotenoid beta-carotene to manipulate skin colour found that beta-carotene supplementation increased skin yellowness and redness to enhance attractiveness within individuals^31^. In terms of variation across individuals, only two studies have examined the relationship using natural, un-manipulated images in men^21^,^35^ and only one study has done so in women^41^. Scott *et al*.^35^ found that skin yellowness positively predicted attractiveness in British Caucasian men (*N* = 75). Stephen *et al*.^21^ found that yellowness positively predicted attractiveness in both British Caucasian (*N* = 34) and African (*N* = 41) men. They also found that lightness positively predicted attractiveness in the British Caucasian men and had a curvilinear relationship with attractiveness in the African men. In African women, Coetzee *et al*.^41^ found that attractiveness was positively related to a skin colour principal component that indicated lighter, yellower and redder skin colour (*N* = 45). Using a larger sample of Australian Caucasians (101 men and 80 women), we found no evidence to support the findings of these three previous correlational studies. Collectively, the results from the literature suggest that skin colour may well influence attractiveness within individuals. But its effect among individuals is less consistent. Given that humans often interact with others on a recurrent basis, one possibility is that skin colour might function as a short-term signal of within-individual condition that allows us to monitor the well-being of individuals that we associate with.

Besides predicting attractiveness, masculinity also positively predicted semen quality. This result is in line with the phenotype-linked fertility hypothesis^13^, which proposes that male secondary sexual traits are attractive because they provide information about male fertility. However, our result is not consistent with those of previous studies. Soler *et al*.^38^ found that the width of the face, a measurement that has been linked to masculinity, was negatively related to semen quality. Similarly, Simmons *et al*.^42^ found that voice masculinity was negatively related to semen quality. In contrast, Peters *et al*.^39^ found no relationship between rated masculinity and semen quality that was quantified manually. Key methodological differences exist between each of these studies and the present one, including how facial masculinity was quantified (i.e. measured *vs* rated; as discussed above), the domain from which masculinity was determined (i.e. voice *vs* face), and how semen quality was measured (i.e. manual *vs* automated). These methodological differences might contribute to the different findings.

In contrast to the positive relationship between masculinity and semen quality, we found a negative relationship between facial averageness and semen quality in men. In general, theories of sexual signalling predict that attractive appearance would be positively related to actual health^10^,^11^,^13^. However, life-history trade-offs can lead to negative relationships between appearance and health, especially in appearance traits that are under good-genes selection^43^,^44^,^45^. From a life history perspective, individuals have limited resources, which they have to allocate to different life history traits^43^. If one allocates more resources to one life history trait, less is available for other traits. Depending on the environment and the individual’s ability to pay the cost of diverting resources from one life history trait to another, trade-offs can lead to either positive or negative relationships between life history traits^44^,^45^. Trade-offs can occur between different aspects of health. An example is the negative relationship between immune function and semen quality in men found in the present study, which is consistent with suggestions that male immune function is traded-off against fertility^46^,^47^. Trade-offs may also occur between health and appearance, which have implications for understanding how attractive traits maintain honesty as signals of health. Animal studies have found negative relationships indicating trade-offs between appearance and health^48^,^49^. The negative relationship between averageness and semen quality that we found could suggest that trade-offs between appearance and health can occur in humans as well.

We did not find any evidence that female attractiveness signalled health. Neither attractiveness nor its components (i.e. femininity and adiposity) were positively related to any of the health variables. Although our results suggest that female attractiveness does not signal immune function or oxidative stress, it might signal other aspects of health, like fertility. Law Smith *et al*.^50^ showed that female facial femininity is positively related to estrogen levels. Increased estrogen levels have been positively associated with the probability of conception in women^51^,^52^. It is possible, therefore, that men are picking up estrogen-related fertility cues in women via femininity.

In recent years, there has been an increased interest regarding the role that carotenoid-based skin yellowness plays in human sexual selection^19^,^20^,^21^. In the present study, we found a negative relationship between skin yellowness and semen quality, which could suggest a trade-off relationship between the two traits. In a previous supplementation study, we found that oral supplementation of the carotenoid beta-carotene did not affect immune function, oxidative stress, or semen quality in humans, suggesting that carotenoids have little impact on human health^31^. Therefore, the negative relationship between skin yellowness and semen quality in the present study might be explained by other unknown factors and not carotenoids. Importantly, we did not find any evidence that skin yellowness predicted attractiveness independent of the other facial traits in either sex. Therefore, it is unlikely that carotenoid-based skin yellowness functions as a sexual signal of health among individuals.

We did not replicate Gangestad *et al*.’s^37^ findings that facial attractiveness and rated masculinity are related to men’s oxidative stress levels. We took steps based on their results to maximise our chances of finding significant results. The urine samples that we used to measure oxidative stress were taken during our afternoon lab sessions, which were shown in Gangestad *et al*.^37^ to have a stronger relationship with facial appearance compared to morning-awakening samples. We also used more than one measure of oxidative stress to obtain a more representative measure of systemic oxidative stress^37^. We note that there was a huge difference in the average oxidative stress levels between our participants and those of Gangestad *et al*.^37^. The mean 8-OHdG levels in our participants were 6.5 ng/mg creatinine for men and 7.7 ng/mg creatinine for women. In contrast, the mean 8-OHdG levels of the male participants in Gangestad *et al*.^37^ were more than 100 times that. We do not know why there was such a huge difference between the two studies, given that the participants from both studies were recruited from similar populations (i.e. relatively young individuals from a university community). The mean 8-OHdG levels in Gangestad *et al*.^37^ were much higher even when compared to published reference values of 8-OHdG levels in healthy individuals, which typically range from 11.9 ng/mg creatinine^53^ to 43.9 ng/mg creatinine^54^. In comparison to these values, the mean 8-OHdG levels in our sample are low. Therefore, it is possible we did not find significant results because all our participants had relatively low oxidative stress levels.

Out of the health measures used in the present study, we only found relationships between appearance and male semen quality. Health is complex and multi-faceted. Although we have used multiple measures of health, especially those that have been theoretically linked to human sexual selection, there are many other aspects of health that could be related to human facial appearance. Some examples that have been examined in other studies include the major histocompatibility complex (MHC) genes^55^, health outcomes (e.g. sickness incidences)^56^,^57^, and longevity^56^. However, mixed results have also been found with such measures^55^,^56^,^57^. Apart from the myriad of possible health measures, the measurement of health is further complicated by potential trade-offs between different aspects of health, such as that between immune function and semen quality, observed in the present study. To gain a more complete understanding of the relationship between appearance and health in humans, future studies could examine other aspects of health that have not been studied, preferably with multiple measures of health.

In summary, sexual dimorphism, symmetry, averageness, and adiposity play important roles in attractiveness. Skin colour, on the other hand, did not directly predict attractiveness in either sex. In terms of actual health, there was no evidence that female attractive appearance signalled health. However, we found support for the phenotype-linked fertility hypothesis that male masculinity signalled semen quality in our sample of men.

## Methods

### Ethics Statement

This research was conducted in accordance with the Declaration of Helsinki and was approved by the Human Ethics Committee at the University of Western Australia (Ethics approval ref. no. RA/4/1/5909). All participants provided written informed consent prior to their participation in the project in accordance with the National Statement on Ethical Conduct in Human Research of the Australian Government National Health and Medical Research Council and the Australian Research Council.

### Participants

One hundred and one Caucasian men (mean age ± *SD* = 20.8 ± 3.6 years, range = 17–35 years) and 80 Caucasian women (mean age ± *SD* = 21.9 ± 4.6 years, range 17–35 years) were recruited from the University of Western Australia community. They received either course credits or travel remuneration. All participants reported being heterosexual. Forty-three women reported being on various forms of hormonal contraception, 36 reported not using any, and one did not report her hormonal contraceptive usage.

### Procedure

Participants first attended a one-hour laboratory session. The sessions were conducted between 12 pm–6 pm to control for potential variations in any of the health measures due to the circadian rhythm. For women who were not using any hormonal contraception, the session was conducted seven to 14 days from the start of their menstruation to control for potential changes in the health variables during to the menstrual cycle. Participants were asked to abstain from eating anything or drinking any flavoured drinks for 1 hour before their session. Participants first collected 10 ml urine in a sample vial for oxidative stress assays. After rinsing their mouths and resting for ~15 minutes, they also collected 5 ml of saliva in a sample vial via passive drool for salivary innate immune function assays. Both samples were stored in a 4 °C fridge upon collection and frozen at −80 °C within 4 hours of collection.

During the resting period between rinsing their mouth and collecting the saliva sample, we took photographs of the participants’ faces and administered a lifestyle questionnaire. Participants’ faces were photographed under standardized lighting. Men were cleanly shaven. Women were not wearing any make-up or artificial tanning. Participants were seated 1.3 m from a Nikon D7000 camera against a grey background with a grey cape draped over them to standardize the colour of their clothing. Spectacles and jewellery were removed. Participants had their hair pulled back with a hairband and were asked to look straight at the camera while adopting a neutral expression with their mouths closed. An X-Rite ColorChecker Classic chart (Grand Rapids, MI, USA) was placed next to the participants’ faces for colour calibration purposes. The images were taken in Nikon’s NEF raw image format and then converted to lossless PNG files^58^.

The questionnaire included items on their dietary and exercise habits, perceived stress levels, recent or long-term medical conditions, and exposure to various toxins, which we used to identify potential confounding lifestyle factors that might influence our health measures.

After the lab session, male participants also collected a sample of their semen for semen quality measurements. They had to abstain from ejaculating for at least two to no more than six days before doing the collection. They collected the sample at home in a sample vial via masturbation while looking at the front view images of four naked women taken from Thornhill and Grammer^59^. The visual stimulation provided by the images is important for producing a normal ejaculate^60^. Participants were asked to deliver the sample to the laboratory for analysis within one hour of collection. As sperm motility is highly sensitive to fluctuations in ambient temperature, participants were asked to wrap the sample vial using a piece of aluminium foil and maintain its temperature by keeping the vial under their arms or between their legs during delivery. Participants also completed an ejaculate questionnaire, which contained questions about the time of the collection, the percentage of ejaculate collected and the portions lost (initial, middle, end), the number of days since the last ejaculation and the amount of time taken to collect the sample.

### Preparation of images

All images were colour-calibrated using the program Psychomorph^61^ to control for subtle random variations in colour due to lighting and photographic conditions. The program adjusts the colour of the images by comparing the CIELab values of the ColorChecker patches in the images to known values. We then rotated and aligned the faces so that the eyes were all sitting at the same height on a horizontal plane. A black oval mask was applied to hide most of the hair, ears, and neck. The masking procedure is widely used in facial perception studies^62^,^63^,^64^,^65^,^66^.

### Face ratings

An additional 127 Caucasian men (mean age ± *SD* = 32.6 ± 8.0 years, range = 19–49 years) and 131 Caucasian women (mean age ± *SD* = 31.8 ± 7.5 years, range = 18–49 years) were recruited online via Amazon Mechanical Turk to provide opposite-sex ratings for the participants’ faces. One potential issue with using online raters in studies involving skin colour is that online raters are using computer screens that have not been colour-calibrated, which might introduce additional noise to the colour of the presented images. However, studies on facial preferences and ratings, including those on skin colour, have been done using online samples^67^,^68^,^69^. One study showed that ratings of attractiveness in faces that varied in skin colour did not differ between raters who were tested in the laboratory using a colour-calibrated monitor versus those who were tested online using their own computers^68^. This result suggests that face ratings from online samples are comparable to that from laboratory samples.

Each rater was randomly assigned to provide opposite-sex ratings on one of the following: attractiveness, perceived health, sexual dimorphism (masculinity for male faces and femininity for female faces), averageness, symmetry, and adiposity on a 9-point scale (1 = not at all, 9 = extremely). For averageness, we asked raters to rate the distinctiveness of the opposite-sex faces following previous studies and reverse coded the ratings to obtain a measure of averageness^6^,^9^,^70^. The face images were cropped and presented at 372 × 491 pixels at a resolution of 72 pixels/in. Each face remained onscreen until the rater provided a response. The number of opposite-sex raters for each trait ranged from 16 to 31. With the exception of male ratings of female averageness, which had a moderate Cronbach’s alpha of 0.53, the inter-rater reliability of all ratings was high, with a Cronbach’s alpha range of 0.74 to 0.96 (see Table S4 for details). For each trait, we calculated an average rating for each face by averaging across raters.

### Face colour measurements

We used the program ImageJ ( http://imagej.nih.gov/ij/) to measure facial skin colour from the colour-calibrated images. The colour measurements were based on ten 60 × 60 pixel skin patches. Four patches were taken from the forehead and six were taken from either side of the cheek. Previous studies have measured facial skin colour from similar regions^20^. Patches were taken from regions without blemishes, shadows, or specular highlights. Average RGB values were extracted from each patch and converted to CIELab values using the equations from the website EasyRGB ( http://www.easyrgb.com/index.php?X=MATH). The CIELab colour space has been used in previous studies on carotenoid-based skin yellowness^19^,^20^,^21^. It contains three colour axes, namely lightness, redness, and yellowness, which approximate the human colour visual system. The CIELab values were averaged across the ten patches to form average lightness, redness, and yellowness values for each face. The CIELab values were moderately repeatable across the 10 squares (lightness L*: *R* = 0.59, 95% CI [0.54, 0.65]; redness a*: *R* = 0.65, 95% CI [0.60, 0.70]; yellowness b*: *R* = 0.64, 95% CI [0.59, 0.69]).

### Immune function assays

We measured salivary innate immune function using two measures. First, we measured salivary antibacterial capacity against *Escherichia coli*^71^. Supernatant from each sample was diluted 2:1 using CO_2_-independent media containing 4 mM L-glutamine and incubated with *E. coli* (ATCC no. 8739) for 30 minutes for bacteria killing to occur. The mixtures were then plated overnight on trypticase soy agar in triplicates. Positive control plates were prepared using the same procedure with bacteria that was diluted with media alone. The concentration of the *E. coli* was adjusted such that there would be ~100–300 colonies on the positive control plates. Images of the plates were taken with a ruler as a size reference. We quantified the salivary bacteria killing capacity based on the percentage of colonies killed relative to the positive controls, the salivary bacteria growth suppression capacity based on the percentage reduction in average colony size relative to the positive controls, and the overall salivary bacterial immunity based on the percentage reduction in total bacteria area on the plate relative to positive controls. The number of colonies, average colony area, and total colony area were highly repeatable (colony number: *R* = 0.95, 95% CI [0.94, 0.96]; average colony area: *R* = 0.90, 95% CI [0.88, 0.93]; total colony area: *R* = 0.95, 95% CI [0.94, 0.96]).

Second, we measured salivary lysozyme activity against *Micrococcus lysodekticus* (ATCC no. 4698) in duplicates. Powdered *M. lysodekticus* was reconstituted using phosphate buffer saline (PBS) to form a cloudy suspension. Ten microliters of *M. lysodekticus* was added to 80 μl of whole saliva in a 96-well plate. Positive controls were created using *M. lysodekticus* and PBS. The plate was incubated for 10 minutes at 33 °C and the resultant absorbance was measured using a M5 SpectraMax microplate reader (Molecular Devices, Sunnyvale, CA). Lysozyme activity was calculated as the difference in absorbance in the sample wells relative to the positive controls. The absorbance was highly repeatable (*R* = 0.96, 95% CI [0.95, 0.97]).

### Oxidative stress

We measured the level of oxidative DNA damage and the level of lipid peroxidation by quantifying the urinary 8-OHdG and isoprostane levels, respectively, using enzyme-linked immunosorbent assay kits (Northwest Life Science Specialties, Vancouver, VA, USA). For the isoprostane assay, the urine samples were pre-treated with beta-glucuronidase before we ran the assay. The pre-treatment frees isoprostanes that are bound to glucuronic acid in the urine, thus giving us a more accurate measure of systemic lipid peroxidation^72^. Both assays were run in duplicates. We also measured urine concentration in duplicates using colorimetric assays (Northwest Life Science Specialties, Vancouver, VA, USA). We standardized both the 8-OHdG and isoprostane results against urine concentration by expressing the results in terms of ng/mg creatinine. The 8-OHdG, isoprostane, and creatinine assays were highly repeatable (8-OHdG: *R* = 0.99, 95% CI [0.98, 0.99]; isoprostane: *R* = 0.94, 95% CI [0.92, 0.96]; creatinine: *R* = 1.00, 95% CI [1.00, 1.00]).

### Semen quality

Semen quality was measured using a Hamilton Thorne computer aided sperm analysis (CASA) system immediately upon delivery of each sample. Each sample was loaded into a Leja four-chamber semen analysis slide (3 μl per chamber). The slide was left on a 37 °C warming stage for 2 minutes before we ran the analyses. Six replicate measurements were taken for each sample. The CASA system measures sperm concentration, percentage motile sperm, and seven motility-related variables (Table S2). Nine samples had to be diluted because they were too concentrated for the CASA to analyse. Each sample was diluted 3:1 using its own seminal fluid, which was extracted by centrifuging a portion of the sample at 12470 × g for 5 minutes. The diluted samples were gently pipetted several times to ensure proper mixing before we ran the analysis. We also took note of whether each sample was completely liquified and whether there were any observed abnormalities with each sample when viewed under the microscope (e.g. regions of dead sperms).

## Additional Information

**How to cite this article**: Foo, Y. Z. *et al*. Predictors of facial attractiveness and health in humans. *Sci. Rep.* **6**, 39731; doi: 10.1038/srep39731 (2016).

**Publisher's note:** Springer Nature remains neutral with regard to jurisdictional claims in published maps and institutional affiliations.

## Supplementary Material

Supplementary Results and Tables

## Figures and Tables

**Table 1:** Zero-order Pearson’s correlations between facial appearance and health, with the corresponding *p*-values and sample sizes.

	Age	Attractiveness	Perceived health	Sexual dimorphism*	Averageness	Symmetry	Adiposity	Lightness	Redness	Yellowness	8OHdG	Isoprostane	Immune PC1	Immune PC2	Semen PC1	Semen PC2	Semen PC3
Age	—	−0.27	−0.25	−0.32	0.01	−0.27	−0.05	−0.17	0.18	0.20	−0.02	0.12	0.05	−0.15	—	—	—
		0.01	0.03	0.004	0.91	0.02	0.63	0.14	0.11	0.07	0.86	0.28	0.64	0.20			
		79	79	79	79	79	79	80	80	80	78	80	77	78			
Attractiveness	−0.08	—	0.76	0.87	−0.01	0.57	−0.58	0.09	−0.09	0.02	−0.19	0.18	−0.36	0.12	—	—	—
	0.41		<0.001	<0.001	0.94	<0.001	<0.001	0.46	0.45	0.88	0.09	0.12	0.002	0.32			
	100		79	79	79	79	79	79	79	79	77	79	76	77			
Perceived health	−0.11	0.84	−	0.74	−0.18	0.53	−0.40	0.06	−0.10	0.00	−0.15	0.22	−0.27	0.03	—	—	—
	0.30	<0.001		<0.001	0.12	<0.001	<0.001	0.62	0.38	0.98	0.18	0.05	0.02	0.76			
	100	101		79	79	79	79	79	79	79	77	79	76	77			
Sexual dimorphism*	0.43	0.30	0.15	−	−0.06	0.58	−0.51	0.14	−0.23	0.07	−0.19	0.14	−0.24	0.13	—	—	—
	<0.001	0.003	0.14		0.62	<0.001	<0.001	0.23	0.04	0.53	0.10	0.22	0.03	0.25			
	100	101	101		79	79	79	79	79	79	77	79	76	77			
Averageness	−0.02	0.33	0.30	−0.13	—	0.06	0.12	−0.06	0.11	−0.12	−0.05	−0.15	0.14	0.11	—	—	—
	0.83	0.001	0.002	0.19		0.61	0.28	0.59	0.34	0.29	0.68	0.18	0.23	0.33			
	100	101	101	101		79	79	79	79	79	77	79	76	77			
Symmetry	−0.03	0.36	0.41	0.12	0.07	—	−0.26	0.00	0.01	−0.01	−0.18	0.05	−0.24	0.14	—	—	—
	0.80	<0.001	<0.001	0.23	0.50		0.02	0.97	0.92	0.93	0.11	0.69	0.03	0.23			
	100	101	101	101	101		79	79	79	79	77	79	76	77			
Adiposity	0.25	−0.50	−0.56	0.02	0.16	−0.01	—	−0.01	0.19	−0.21	0.09	−0.08	0.17	0.04	—	—	—
	0.01	<0.001	<0.001	0.85	0.11	0.93		0.93	0.10	0.07	0.45	0.49	0.13	0.75			
	100	101	101	101	101	101		79	79	79	77	79	76	77			
Lightness	−0.07	−0.28	−0.22	−0.11	0.11	−0.23	−0.03	—	−0.65	−0.35	0.00	0.24	−0.15	−0.03	—	—	—
	0.47	0.01	0.03	0.29	0.28	0.02	0.76		<0.001	0.001	0.97	0.03	0.18	0.78			
	100	101	101	101	101	101	101		80	80	78	80	77	78			
Redness	0.00	0.17	0.09	0.10	0.06	0.14	0.14	−0.62	—	−0.07	−0.07	−0.06	0.15	−0.02	—	—	—
	0.97	0.09	0.36	0.31	0.54	0.16	0.18	<0.001		0.55	0.54	0.62	0.20	0.87			
	100	101	101	101	101	101	101	101		80	78	80	77	78			
Yellowness	0.12	0.23	0.26	0.05	−0.03	0.22	−0.18	−0.32	−0.11	—	−0.05	−0.15	0.01	0.10	—	—	—
	0.24	0.02	0.01	0.62	0.78	0.03	0.08	0.001	0.28		0.67	0.19	0.96	0.38			
	100	101	101	101	101	101	101	101	101		78	80	77	78			
8OHdG	−0.03	0.20	0.21	0.07	0.09	−0.01	−0.22	−0.10	0.04	0.02	—	−0.15	0.06	−0.38	—	—	—
	0.80	0.05	0.04	0.51	0.37	0.93	0.03	0.32	0.67	0.85		0.18	0.58	<0.001			
	97	98	98	98	98	98	98	98	98	98		78	76	76			
Isoprostane	0.23	0.14	0.12	0.17	0.09	0.05	0.03	−0.04	0.13	−0.01	0.03	—	−0.34	0.15	—	—	—
	0.02	0.17	0.23	0.08	0.35	0.60	0.76	0.71	0.21	0.92	0.77		0.002	0.18			
	100	101	101	101	101	101	101	101	101	101	98		77	78			
Immune PC1	0.11	−0.16	−0.14	−0.07	0.01	0.10	0.20	−0.04	0.00	−0.03	−0.02	0.44	—	−0.07	—	—	—
	0.28	0.11	0.17	0.47	0.95	0.32	0.05	0.69	1.00	0.73	0.84	<0.001		0.53			
	97	98	98	98	98	98	98	98	98	98	95	98		77			
Immune PC2	−0.16	0.04	0.01	−0.14	0.11	−0.08	−0.15	0.13	0.02	−0.04	−0.10	−0.13	0.00	—	—	—	—
	0.11	0.72	0.92	0.16	0.29	0.42	0.15	0.19	0.83	0.73	0.32	0.20	1.00				
	97	98	98	98	98	98	98	98	98	98	95	98	98				
Semen PC1	−0.11	0.01	0.05	−0.10	−0.05	−0.15	−0.01	0.10	−0.07	−0.30	−0.01	0.05	0.20	−0.03	—	—	—
	0.33	0.95	0.67	0.34	0.62	0.16	0.90	0.35	0.54	0.01	0.96	0.67	0.07	0.79			
	87	87	87	87	87	87	87	87	87	87	84	87	84	84			
Semen PC2	0.21	0.18	0.11	0.29	−0.05	−0.01	−0.06	−0.23	0.22	0.13	0.05	0.09	−0.07	0.09	−0.33	—	—
	0.06	0.09	0.29	0.01	0.65	0.93	0.60	0.03	0.04	0.22	0.63	0.39	0.55	0.42	0.002		
	86	86	86	86	86	86	86	86	86	86	83	86	84	84	86		
Semen PC3	0.21	0.01	0.03	0.23	−0.21	0.05	−0.04	0.01	−0.10	0.10	−0.14	0.09	0.03	−0.23	0.09	0.01	—
	0.05	0.95	0.78	0.03	0.04	0.62	0.69	0.93	0.35	0.34	0.20	0.37	0.81	0.03	0.43	0.92	
	91	91	91	91	91	91	91	91	91	91	88	91	88	88	87	86	

Male results are below the diagonal. Female results are above the diagonal.

^*^Masculinity for men and femininity for women.

**Table 2:** Means and *SD*s of the face ratings (rated on a 9-point scale) and the three skin colour measurements based on the CIELab colour space.

	Men		Women	
	*M*	(*SD*)	*M*	(*SD*)
Attractiveness	3.33	(0.90)	3.46	(0.83)
Perceived health	5.26	(1.07)	5.16	(0.72)
Sexual dimorphism*	5.64	(0.91)	4.81	(0.97)
Averageness	5.54	(0.81)	5.16	(0.47)
Symmetry	5.07	(0.76)	5.12	(0.72)
Adiposity	5.46	(0.92)	5.46	(0.82)
Skin lightness	63.70	(2.58)	64.28	(2.35)
Skin redness	13.42	(1.95)	12.27	(1.76)
Skin yellowness	20.34	(1.94)	20.24	(1.99)

*Masculinity in men, femininity in women.

**Table 3:** Facial appearance predictors of facial attractiveness.

	Men						Women					
	B ± SE	β	*t*(df)	*p*	*r*	95% CI	B ± SE	β	*t*(df)	*p*	*r*	95% CI
**Regression 1**												
Age	−0.03 ± 0.02	−0.12	−1.43 (94)	0.16	−0.15	[−0.33, 0.05]	−0.01 ± 0.01	−0.03	−0.58 (73)	0.56	−0.07	[−0.28, 0.16]
Sexual dimorphism	0.35 ± 0.08	0.35	4.33 (94)	<0.001	0.41	[0.23, 0.56]	0.60 ± 0.07	0.70	8.91 (73)	<0.001	0.72	[0.60, 0.81]
Averageness	0.31 ± 0.08	0.28	3.88 (94)	<0.001	0.37	[0.19, 0.53]	0.09 ± 0.10	0.05	0.95 (73)	0.35	0.11	[−0.11, 0.32]
Symmetry	0.36 ± 0.09	0.30	4.13 (94)	<0.001	0.39	[0.21, 0.55]	0.12 ± 0.08	0.10	1.50 (73)	0.14	0.17	[−0.05, 0.38]
Adiposity	−0.41 ± 0.07	−0.42	−5.64 (94)	<0.001	−0.50	[−0.64, −0.34]	−0.21 ± 0.07	−0.21	−3.17 (73)	0.00	−0.35	[−0.53, −0.14]
Adiposity^−2^	—	—	—	—	—	—	0.06 ± 0.06	0.71	1.07 (72)	0.29	0.12	[−0.10, 0.34]
**Regression 2**												
Age	−0.03 ± 0.02	−0.12	−1.22 (95)	0.22	−0.12	[−0.31, 0.07]	−0.05 ± 0.02	−0.29	−2.48 (74)	0.02	−0.28	[−0.47, −0.06]
Lightness	−0.05 ± 0.05	−0.14	−0.99 (95)	0.33	−0.10	[−0.29, 0.10]	0.04 ± 0.06	0.11	0.62 (74)	0.54	0.07	[−0.15, 0.29]
Redness	0.06 ± 0.07	0.12	0.86 (95)	0.39	0.09	[−0.11, 0.28]	0.02 ± 0.08	0.04	0.26 (74)	0.80	0.03	[−0.19, 0.25]
Yellowness	0.10 ± 0.05	0.21	1.83 (95)	0.07	0.18	[−0.01, 0.37]	0.05 ± 0.06	0.11	1.86 (74)	0.39	0.10	[−0.12, 0.31]

Separate multiple regression models were conducted for attractive traits skin colour and other potential components of attractiveness (i.e. sexual dimorphism, averageness, symmetry, and adiposity).

**Table 4:** Facial appearance predictors of perceived health.

	Men						Women					
	B ± SE	β	*t*(df)	*p*	*r*	95% CI	B ± SE	β	*t*(df)	*p*	*r*	95% CI
**Regression 1**												
Age	−0.01 ± 0.02	−0.03	−0.33 (94)	0.74	−0.03	[−0.23, 0.16]	0.00 ± 0.01	0.01	0.07 (73)	0.95	0.01	[−0.21, 0.23]
Sexual dimorphism	0.17 ± 0.09	0.15	1.84 (94)	0.07	0.19	[−0.01, 0.37]	0.46 ± 0.08	0.63	5.76 (73)	<0.001	0.56	[0.39, 0.69]
Averageness	0.29 ± 0.10	0.22	3.04 (94)	0.00	0.30	[0.11, 0.47]	−0.23 ± 0.12	−0.15	−1.98 (73)	0.05	−0.23	[−0.43, −0.01]
Symmetry	0.55 ± 0.10	0.38	5.38 (94)	<0.001	0.49	[0.32, 0.62]	0.18 ± 0.09	0.18	1.88 (73)	0.06	0.22	[−0.01, 0.42]
Adiposity	−0.60 ± 0.09	−0.51	−6.92 (94)	<0.001	−0.58	[−0.70 −0.43]	−0.01 ± 0.08	−0.01	−0.10 (73)	0.92	−0.01	[−0.23, 0.21]
**Regression 2**												
Age	−0.04 ± 0.03	−0.14	−1.46 (95)	0.15	−0.15	[−0.33, 0.05]	−0.04 ± 0.02	−0.25	−2.10 (74)	0.04	−0.24	[−0.44, − 0.02]
Lightness	−0.05 ± 0.06	−0.12	−0.79 (95)	0.43	−0.08	[−0.27, 0.12]	0.00 ± 0.05	0.00	−0.02 (74)	0.99	0.00	[−0.22, 0.22]
Redness	0.03 ± 0.08	0.06	0.43 (95)	0.67	0.04	[−0.15, 0.24]	−0.02 ± 0.07	−0.06	−0.34 (74)	0.74	−0.04	[−0.26, 0.18]
Yellowness	0.13 ± 0.06	0.24	2.14 (95)	0.03	0.21	[0.02, 0.39]	0.02 ± 0.05	0.05	0.34 (74)	0.73	0.04	[−0.18, 0.26]

Separate multiple regression models were conducted for skin colour and other potential components of attractiveness (i.e. sexual dimorphism, averageness, symmetry, and adiposity).

**Table 5:** Facial appearance predictors of immune function PC1.

	Men						Women					
	B ± SE	β	*t*(df)	*p*	*r*	95% CI	B ± SE	β	*t*(df)	*p*	*r*	95% CI
**Regression 1**												
Age	0.04 ± 0.03	0.14	1.18 (91)	0.24	0.12	[−0.08, 0.31]	−0.01 ± 0.03	−0.03	−0.25 (70)	0.80	−0.03	[−0.25, 0.20]
Sexual dimorphism	−0.17 ± 0.13	−0.15	−1.28 (91)	0.20	−0.13	[−0.32, 0.07]	−0.12 ± 0.17	−0.12	−0.72 (70)	0.48	−0.09	[−0.30, 0.14]
Averageness	0.00 ± 0.13	0.00	0.03 (91)	0.98	0.00	[−0.20, 0.20]	0.30 ± 0.24	0.14	1.23 (70)	0.22	0.15	[−0.08, 0.36]
Symmetry	0.16 ± 0.14	0.12	1.21 (91)	0.23	0.13	[−0.08, 0.32]	−0.25 ± 0.20	−0.18	−1.29 (70)	0.20	−0.15	[−0.37, 0.08]
Adiposity	0.18 ± 0.12	0.17	1.56 (91)	0.12	0.16	[−0.04, 0.35]	0.06 ± 0.17	0.05	0.38 (70)	0.70	0.05	[−0.18, 0.27]
**Regression 2**												
Age	0.03 ± 0.03	0.11	1.07 (92)	0.29	0.11	[−0.09, 0.30]	0.01 ± 0.03	0.03	0.25 (71)	0.80	0.03	[−0.20, 0.25]
Lightness	−0.05 ± 0.06	−0.13	−0.82 (92)	0.41	−0.09	[−0.28, 0.12]	−0.06 ± 0.08	−0.13	−0.70 (71)	0.49	−0.08	[−0.30, 0.15]
Redness	−0.05 ± 0.08	−0.10	−0.69 (92)	0.49	−0.07	[−0.27, 0.13]	0.03 ± 0.10	0.05	0.31 (71)	0.76	0.04	[−0.19, 0.26]
Yellowness	−0.05 ± 0.06	−0.10	−0.83 (92)	0.41	−0.09	[−0.28, 0.12]	−0.02 ± 0.07	−0.05	−0.34 (71)	0.74	−0.04	[−0.26, 0.19]

Separate multiple regression models were conducted for skin colour and other potential components of attractiveness (i.e. sexual dimorphism, averageness, symmetry, and adiposity).

**Table 6:** Facial appearance predictors of immune function PC2.

	Men						Women					
	B ± SE	β	*t*(df)	*p*	*r*	95% CI	B ± SE	β	*t*(df)	*p*	*r*	95% CI
**Regression 1**												
Age	−0.03 ± 0.03	−0.11	−0.93 (91)	0.36	−0.10	[−0.29, 0.10]	−0.02 ± 0.03	−0.09	−0.73 (71)	0.47	−0.09	[−0.30, 0.14]
Sexual dimorphism	−0.08 ± 0.13	−0.07	−0.60 (91)	0.55	−0.06	[−0.26, 0.14]	0.13 ± 0.17	0.13	0.74 (71)	0.46	0.09	[−0.14, 0.31]
Averageness	0.10 ± 0.13	0.08	0.80 (91)	0.43	0.08	[−0.12, 0.28]	0.23 ± 0.25	0.11	0.91 (71)	0.37	0.11	[−0.12, 0.32]
Symmetry	−0.12 ± 0.14	−0.09	−0.87 (91)	0.39	−0.09	[−0.29, 0.11]	0.09 ± 0.20	0.06	0.44 (71)	0.66	0.05	[−0.17, 0.27]
Adiposity	−0.12 ± 0.12	−0.11	−1.07 (91)	0.29	−0.11	[−0.30, 0.09]	0.13 ± 0.17	0.10	0.74 (71)	0.47	0.09	[−0.14, 0.30]
**Regression 2**												
Age	−0.04 ± 0.03	−0.15	−1.48 (92)	0.14	−0.15	[−0.34, 0.05]	−0.04 ± 0.03	−0.18	−1.48 (72)	0.14	−0.17	[−0.38, 0.05]
Lightness	0.11 ± 0.06	0.29	1.88 (92)	0.06	0.19	[−0.01, 0.38]	0.01 ± 0.08	0.01	0.07 (72)	0.94	0.01	[−0.22, 0.23]
Redness	0.11 ± 0.08	0.21	1.44 (92)	0.15	0.15	[−0.05, 0.34]	0.01 ± 0.10	0.03	0.15 (72)	0.88	0.02	[−0.21, 0.24]
Yellowness	0.05 ± 0.06	0.10	0.84 (92)	0.41	0.09	[−0.11, 0.28]	0.07 ± 0.07	0.13	0.98 (72)	0.33	0.11	[−0.11, 0.33]

Separate multiple regression models were conducted for skin colour and other potential components of attractiveness (i.e. sexual dimorphism, averageness, symmetry, and adiposity).

**Table 7:** Means and *SD*s (in ng/mg creatinine) and multiple regression models on the facial appearance predictors of 8-OHdG levels.

	*M(SD*)	Men						*M(SD*)	Women					
		B ± SE	β	*t*(df)	*p*	*r*	95% CI		B ± SE	β	*t*(df)	*p*	*r*	95% CI
	6.45 (3.23)							7.67 (3.70)						
**Regression 1**														
Age		−0.02 ± 0.11	−0.02	−0.18 (91)	0.86	−0.02	[−0.22, 0.18]		−0.02 ± 0.03	−0.11	−0.87 (71)	0.39	−0.10	[−0.32, 0.12]
Sexual dimorphism		0.32 ± 0.42	0.09	0.78 (91)	0.44	0.08	[−0.12, 0.28]		−0.18 ± 0.17	−0.17	−1.05 (71)	0.30	−0.12	[−0.34, 0.10]
Averageness		0.29 ± 0.41	0.07	0.69 (91)	0.49	0.07	[−0.13, 0.27]		−0.09 ± 0.24	−0.04	−0.39 (71)	0.70	−0.05	[−0.27, 0.18]
Symmetry		−0.07 ± 0.45	−0.02	−0.16 (91)	0.88	−0.02	[−0.22, 0.18]		−0.16 ± 0.19	−0.12	−0.82 (71)	0.42	−0.10	[−0.31, 0.13]
Adiposity		−0.70 ± 0.38	−0.20	−1.87 (91)	0.06	−0.19	[−0.38, 0.01]		−0.03 ± 0.17	−0.03	−0.19 (71)	0.85	−0.02	[−0.25, 0.20]
**Regression 2**														
Age		−0.03 ± 0.10	−0.03	−0.30 (92)	0.76	−0.03	[−0.23, 0.17]		0.00 ± 0.03	0.01	0.06 (72)	0.95	0.01	[−0.22, 0.23]
Lightness		−0.16 ± 0.20	−0.13	−0.82 (92)	0.42	−0.09	[−0.28, 0.12]		−0.07 ± 0.07	−0.17	−0.92 (72)	0.36	−0.11	[−0.32, 0.12]
Redness		−0.04 ± 0.25	−0.02	−0.16 (92)	0.87	−0.02	[−0.22, 0.18]		−0.11 ± 0.10	−0.19	−1.11 (72)	0.27	−0.13	[−0.34, 0.10]
Yellowness		−0.04 ± 0.20	−0.02	−0.18 (92)	0.86	−0.02	[−0.22, 0.18]		−0.06 ± 0.07	−0.13	−0.89 (72)	0.38	−0.10	[−0.32, 0.12]

Separate multiple regression models were conducted for skin colour and other potential components of attractiveness (i.e. sexual dimorphism, averageness, symmetry, and adiposity).

**Table 8:** Means and *SD*s (in ng/mg creatinine) and multiple regression models on the facial appearance predictors of 8-isoprostane levels.

	*M(SD*)	Men						*M(SD*)	Women					
		B ± SE	β	*t*(df)	*p*	*r*	95% CI		B ± SE	β	*t*(df)	*p*	*r*	95% CI
	2.28 (1.60)							1.91 (1.37)						
**Regression 1**														
Age		0.02 ± 0.01	0.19	1.68 (94)	0.10	0.17	[−0.03, 0.36]		0.06 ± 0.04	0.19	1.52 (73)	0.13	0.18	[−0.05, 0.38]
Sexual dimorphism		0.03 ± 0.04	0.09	0.81 (94)	0.42	0.08	[−0.12, 0.28]		0.33 ± 0.23	0.24	1.43 (73)	0.16	0.16	[−0.06, 0.37]
Averageness		0.04 ± 0.04	0.11	1.12 (94)	0.27	0.11	[−0.08, 0.30]		−0.44 ± 0.33	−0.15	−1.32 (73)	0.19	−0.15	[−0.36, 0.07]
Symmetry		0.03 ± 0.04	0.06	0.63 (94)	0.53	0.06	[−0.13, 0.26]		−0.03 ± 0.27	−0.02	−0.11 (73)	0.91	−0.01	[−0.23, 0.21]
Adiposity		0.00 ± 0.04	0.01	0.13 (94)	0.90	0.01	[−0.18, 0.21]		0.11 ± 0.23	0.07	0.49 (73)	0.63	0.06	[−0.17, 0.27]
**Regression 2**														
Age		0.02 ± 0.01	0.24	2.43 (95)	0.02	0.24	[0.05, 0.42]		0.05 ± 0.03	0.16	1.36 (74)	0.18	0.16	[−0.07, 0.36]
Lightness		0.02 ± 0.02	0.16	1.12 (95)	0.27	0.11	[−0.08, 0.30]		0.20 ± 0.10	0.34	1.98 (74)	0.05	0.22	[0.00, 0.42]
Redness		0.05 ± 0.02	0.27	1.93 (95)	0.06	0.19	[0.00, 0.38]		0.09 ± 0.13	0.12	0.75 (74)	0.46	0.09	[−0.14, 0.30]
Yellowness		0.01 ± 0.02	0.04	0.38 (95)	0.71	0.04	[−0.16, 0.23]		−0.04 ± 0.09	−0.06	−0.47 (74)	0.64	−0.05	[−0.27, 0.17]

Separate multiple regression models were conducted for skin colour and other potential components of attractiveness (i.e. sexual dimorphism, averageness, symmetry, and adiposity).

**Table 9:** Male facial appearance predictors of semen PC1.

	B ± SE	β	*t*(df)	*p*	*r*	95% CI
**Regression 1**						
Age	−0.02 ± 0.03	−0.09	−0.71 (81)	0.48	−0.08	[−0.28, 0.13]
Masculinity	−0.05 ± 0.14	−0.05	−0.39 (81)	0.70	−0.04	[−0.25, 0.17]
Averageness	−0.04 ± 0.14	−0.03	−0.30 (81)	0.76	−0.03	[−0.24, 0.18]
Symmetry	−0.20 ± 0.15	−0.15	−1.31 (81)	0.19	−0.14	[−0.34, 0.07]
Adiposity	0.00 ± 0.13	0.00	0.02 (81)	0.99	0.00	[−0.21, 0.21]
**Regression 2**						
Age	−0.01 ± 0.03	−0.06	−0.54 (82)	0.59	−0.06	[−0.27, 0.15]
Lightness	−0.05 ± 0.06	−0.14	−0.90 (82)	0.37	−0.10	[−0.30, 0.11]
Redness	−0.10 ± 0.08	−0.20	−1.34 (82)	0.18	−0.15	[−0.35, 0.07]
Yellowness	−0.18 ± 0.06	−0.36	−2.87 (82)	0.01	−0.30	[−0.48, −0.10]

Separate multiple regression models were conducted for skin colour and other potential components of attractiveness (i.e. sexual dimorphism, averageness, symmetry, and adiposity).

**Table 10:** Male facial appearance predictors of semen PC2.

	B ± SE	β	*t*(df)	*p*	*r*	95% CI
**Regression 1**						
Age	0.03 ± 0.03	0.12	1.01 (80)	0.31	0.11	[−0.10, 0.32]
Masculinity	0.26 ± 0.13	0.24	2.01 (80)	0.05	0.22	[0.01, 0.41]
Averageness	−0.07 ± 0.14	−0.05	−0.51 (80)	0.61	−0.06	[−0.27, 0.16]
Symmetry	−0.04 ± 0.14	−0.03	−0.25 (80)	0.80	−0.03	[−0.24, 0.19]
Adiposity	−0.12 ± 0.12	−0.10	−0.95 (80)	0.35	−0.11	[−0.31, 0.11]
**Regression 2**						
Age	0.05 ± 0.03	0.19	1.81 (81)	0.07	0.20	[−0.02, 0.39]
Lightness	−0.03 ± 0.06	−0.07	−0.43 (81)	0.67	−0.05	[−0.26, 0.17]
Redness	0.10 ± 0.08	0.19	1.26 (81)	0.21	0.14	[−0.08, 0.34]
Yellowness	0.05 ± 0.06	0.10	0.79 (81)	0.43	0.09	[−0.13, 0.29]

Separate multiple regression models were conducted for skin colour and other potential components of attractiveness (i.e. sexual dimorphism, averageness, symmetry, and adiposity).

**Table 11:** Male facial appearance predictors of semen PC3.

	B ± SE	β	*t*(df)	*p*	*r*	95% CI
**Regression 1**						
Age	0.05 ± 0.03	0.18	1.57 (85)	0.12	0.17	[−0.04, 0.36]
Masculinity	0.16 ± 0.13	0.14	1.23 (85)	0.22	0.13	[−0.08, 0.33]
Averageness	−0.31 ± 0.13	−0.24	−2.34 (85)	0.02	−0.25	[−0.43, −0.04]
Symmetry	0.09 ± 0.14	0.07	0.67 (85)	0.50	0.07	[−0.14, 0.27]
Adiposity	−0.15 ± 0.12	−0.13	−1.24 (85)	0.22	−0.13	[−0.33, 0.07]
**Regression 2**						
Age	0.05 ± 0.03	0.20	1.85 (86)	0.07	0.20	[−0.01, 0.39]
Lightness	−0.02 ± 0.06	−0.04	−0.24 (86)	0.81	−0.03	[−0.23, 0.18]
Redness	−0.06 ± 0.08	−0.12	−0.76 (86)	0.45	−0.08	[−0.28, 0.13]
Yellowness	0.02 ± 0.07	0.04	0.32 (86)	0.75	0.03	[−0.17, 0.24]

Separate multiple regression models were conducted for skin colour and other potential components of attractiveness (i.e. sexual dimorphism, averageness, symmetry, and adiposity).
